# The role of sigma factor RpoH1 in the pH stress response of *Sinorhizobium meliloti*

**DOI:** 10.1186/1471-2180-10-265

**Published:** 2010-10-18

**Authors:** Daniella KC de Lucena, Alfred Pühler, Stefan Weidner

**Affiliations:** 1 Lehrstuhl für Genetik, Fakultät für Biologie, Universität Bielefeld, 33594 Bielefeld, Germany; 2 Center for Biotechnology, Universität Bielefeld, 33594 Bielefeld, Germany

## Abstract

**Background:**

Environmental pH stress constitutes a limiting factor for *S. meliloti *survival and development. The response to acidic pH stress in *S. meliloti *is versatile and characterized by the differential expression of genes associated with various cellular functions. The purpose of this study was to gain detailed insight into the participation of sigma factors in the complex stress response system of *S. meliloti *1021 using pH stress as an effector.

**Results:**

*In vitro *assessment of *S meliloti *wild type and sigma factor mutants provided first evidence that the sigma factor RpoH1 plays a major role in the pH stress response. Differential expression of genes related to rhizobactin biosynthesis was observed in microarray analyses performed with the *rpoH1 *mutant at pH 7.0. The involvement of the sigma factor RpoH1 in the regulation of *S. meliloti *genes upon pH stress was analyzed by comparing time-course experiments of the wild type and the *rpoH1 *mutant. Three classes of *S. meliloti *genes could be identified, which were transcriptionally regulated in an RpoH1-independent, an RpoH1-dependent or in a complex manner. The first class of *S. meliloti *genes, regulated in an RpoH1-independent manner, comprises the group of the exopolysaccharide I biosynthesis genes and also the group of genes involved in motility and flagellar biosynthesis. The second class of *S. meliloti *genes, regulated in an RpoH1-dependent manner, is composed of genes known from heat shock studies, like *ibpA, grpE *and *groEL5*, as well as genes involved in translation like *tufA *and *rplC*. Finally, the third class of *S. meliloti *genes was regulated in a complex manner, which indicates that besides sigma factor RpoH1, further regulation takes place. This was found to be the case for the genes *dctA, ndvA *and *smc01505*.

**Conclusions:**

Clustering of time-course microarray data of *S. meliloti *wild type and sigma factor *rpoH1 *mutant allowed for the identification of gene clusters, each with a unique time-dependent expression pattern, as well as for the classification of genes according to their dependence on RpoH1 expression and regulation. This study provided clear evidence that the sigma factor RpoH1 plays a major role in pH stress response.

## Background

Stress response in bacteria is essential for effective adaptation to changes in the environment, as well as to changes in the bacterial physiological state. This response is mediated by global regulatory mechanisms that operate in an effective method of transcriptional control, with the participation of specialized RNA polymerase subunits, the alternative sigma factors [1]. Bacteria usually display two distinct responses to stress conditions: a response that controls the conditions in the cytoplasm, which is orchestrated by the alternative sigma factor σ^32^, and a response to the conditions in the periplasm, which is orchestrated by the alternative sigma factor σ^E ^[2]. Each response deals with the cellular ability to sense protein folding and other signals, and leads to the activation of proteins such as molecular chaperones, proteases, and regulatory factors, which play an important role in promoting homeostasis under stress conditions [3-5].

The heat shock response is a widespread phenomenon found in all living cells. In bacteria, it is controlled at the transcriptional level by the alternative sigma factor RpoH (σ^32^) [6-8]. In addition to the response to high temperatures, RpoH is known to be involved in the response to pH and oxidative stress [9-11]. The σ^32 ^regulon protects many cytoplasmic molecules and processes, including transcription factors, as well as cytoplasmic membranes and inner membrane proteins [6,8]. In *E. coli*, RpoH controls the expression of about 91 genes [12], including many coding for heat shock proteins, which are important for survival during stress conditions. Among these are the genes encoding chaperones, such as GroEL, GroES, DnaK, DnaJ and GrpE and proteases, like FtsH and Lon [13]. Induction of heat shock proteins represents an important protective mechanism to cope with environmental stress, for these proteins mediate the correct folding and assembly of polypeptides. Major functions of heat shock proteins are to prevent inactivation of cellular proteins, to reactivate once inactivated proteins, and to help degrade non-reparable denatured proteins that accumulate under stress conditions [8].

*Sinorhizobium meliloti *is a Gram-negative α-proteobacterium that establishes root-nodulating, nitrogen fixing, symbiosis with leguminous host plants, such as alfalfa [14-16]. Several important steps in the symbiosis process, like nodule formation and nitrogen fixation, are affected by stress conditions, which might be considered limiting factors. In the soil, variations of temperature, osmolarity, or pH, as well as nutrient starvation, are the stress conditions most frequently faced by rhizobia [17]. Commonly, bacterial genomes contain a single *rpoH *gene, but several α-proteobacteria have more than one *rpoH *homologue. Two *rpoH *genes have been identified in *Brucella melitensis *and *Rhodobacter sphaeroides*, and in the nitrogen-fixing symbionts *Mesorhizobium loti *and *Rhizobium etli *[9,11,18,19]; *Bradyrhizobium japonicum *possesses three *rpoH*-like genes [20]. The *S. meliloti *1021 genome contains 14 genes for sigma factors [21], two of which code for RpoH sigma factors. However, *rpoH1 *and *rpoH2 *are not functionally equivalent [22,23]. The two genes are expressed differentially during growth in culture and during symbiosis, and only *rpoH1 *is required for growth in heat shock stress and for successful symbiosis with alfalfa [23,24]. The presence of several copies of RpoH sigma factors suggests that rhizobia may respond more specifically to environmental changes and that the heat shock response could overlap the response to other stimuli [23]. Previous studies with *S. meliloti *revealed that an *rpoH1 *mutant exhibits increased sensitivity to various stress agents, including acid pH, suggesting that RpoH1 is required to protect the bacterial cell against environmental stress encountered in solo or within the host [25].

Soil acidity constrains symbiotic nitrogen fixation and affects the exchange of molecular signals between rhizobia and their host, reducing nodulation [26-28]. Environmental pH stress constitutes therefore a limiting factor for *S. meliloti *survival and development, both in the soil and in planta [29]. In a previous study, it was observed that the response to acidic pH stress in *S. meliloti *is versatile and characterized by the differential expression of whole sets of genes associated with various cellular functions, such as exopolysaccharide I biosynthesis and chemotaxis [30]. The purpose of the present study was to gain detailed insight into the complex stress response regulatory system of *S. meliloti *using pH stress as an effector and to verify if specific sigma factors in *S. meliloti *are involved in pH stress response. Our aim was likewise to provide a basis for understanding the molecular mechanisms of sigma factor regulation and identify genes involved in pH stress response whose expression is sigma factor-dependent. Because the regulation of gene expression is a dynamic process, special attention was granted to the characterization of changes in gene expression over time.

## Results

### Identification of sigma factors involved in the pH stress response of *S. meliloti*

**Figure 1:**
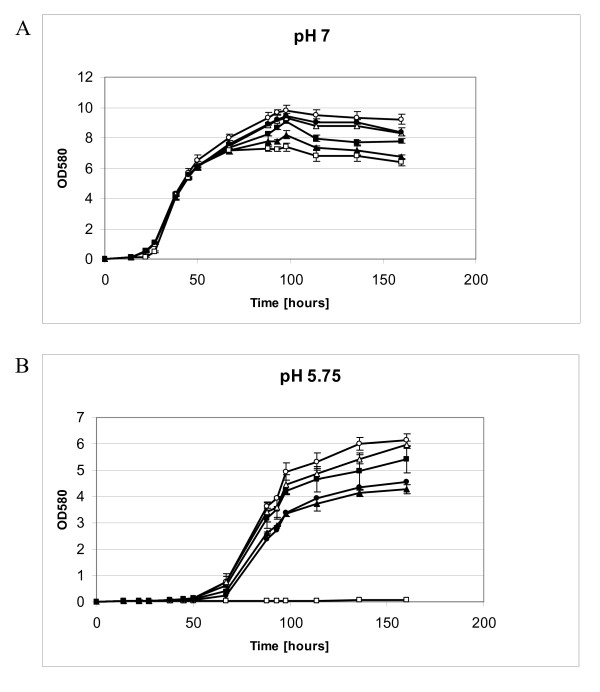
**Growth curves of *S. meliloti *1021 wild type strain and mutant strains for sigma factor genes at neutral and acidic pH**. *S. meliloti *1021 (open circles) and mutant strains for sigma factor genes *rpoE1 *(filled squares), *rpoE2 *(filled triangles), *rpoE5 *(open triangles), *fecI *(filled circles) and *rpoH1 *(open squares) were grown in VMM medium at 30°C at either pH 7.0 (A) or pH 5.75 (B). Each panel shows the data from three representative experiments. The error bars indicate the standard deviation calculated from three independent cultures.

To explore the role of sigma factors in *S. meliloti *under acidic pH stress conditions, marker-free deletion mutants were successfully produced for the sigma factor genes *rpoE1*, *rpoE2*, *rpoE5, rpoH1 *and *fecI*, with the utilization of gene Splicing by Overlap Extension or gene SOEing technique [31]. Those sigma factor genes were chosen for mutant constructions for, based on amino acid sequence comparison analysis, they represent the three main functional classes of alternative sigma factors, namely extracytoplasmic function, heat shock and iron metabolism control. In order to determine the growth properties and to test for a role of those sigma factors in pH stress response, the growth of sigma factor mutant and wild type cells in VMM medium was monitored at two distinct pH values: pH 7.0 and pH 5.75. All sigma factor mutants grew slightly more poorly than wild type cells at both pH 7.0 and pH 5.75, with the exception of the *rpoH1 *mutant, whose growth was severely impaired at pH 5.75 (Figure 1). Restoration of the wild type growth phenotype was observed for the *rpoH1 *mutant carrying a recombinant plasmid with the intact *rpoH1 *gene, confirming that the lack of growth was solely caused by the *rpoH1 *mutation (Additional file 1). The results indicate that the RpoH1 sigma factor is therefore essential for growth at acidic pH.

### Transcription profiling of the *rpoH1 *mutant versus wild type at neutral pH reveals RpoH1 involvement only in the regulation of the rhizobactin operon

**Figure 2:**
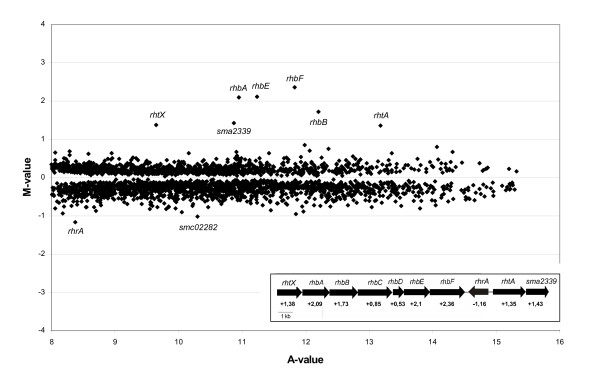
**Scatter plot of the microarray analysis of the *S. meliloti rpoH1 *mutant versus wild type at pH 7.0**. The plot shows the log_2 _ratio (M-value) versus the mean signal intensity (A-value) obtained by comparison of the transcriptomes of *S. meliloti rpoH1 *mutant versus *S. meliloti *wild type strain 1021. Genes with the greatest changes in expression values (-1 ≤ M-value ≥ 1) are indicated. On the low right corner is an illustration of the genetic map for the operon coding for proteins involved in rhizobactin 1021 biosynthesis and uptake. The numbers below the genes indicate the log_2 _expression ratios of the genes obtained through the transcriptome analysis.

Among all the sigma factors analyzed, the *rpoH1 *mutant showed the most peculiar phenotype in the growth tests, presenting no growth at low pH values. This mutant was therefore selected for transcription profiling experiments. With the intent of examining the differential expression of genes in the sigma factor *rpoH1 *deletion mutant in comparison to the wild type, both *S. meliloti *wild type strain 1021 and *rpoH1 *mutant were cultivated at pH 7.0 and harvested for microarray analysis after reaching an optical density of 0.8 at 580 nm. Only genes with a twofold difference in spot intensities on the microarray slides (M-value of ≥ 1 or ≤ -1) were considered. Surprisingly, at neutral pH, the rhizobactin biosynthesis operon was nearly exclusively observed among the significant differentially expressed genes (Figure 2). Rhizobactin is an iron siderophore, that is, a low molecular weight ligand that binds to ferric iron with high affinity [32]. All genes for the rhizobactin biosynthesis operon, *rhbABCDEF*, were upregulated, as well as the rhizobactin transporter gene *rhtA*. The gene for the rhizobactin activator *rhrA*, however, was downregulated in the mutant. The unexpected but dramatic increase in siderophore production by the *rpoH1 *deletion mutant in comparison to the *S. meliloti *wild type was additionally confirmed by Chrome azurol S (CAS) assay, which is a chemical test for the detection of siderophore production based on the removal of ferric iron from a pigmented complex by a competing ligand such as a siderophore [33] (Additional file 2). Except for the genes involved in the rhizobactin siderophore biosynthesis and regulation, basically no other genes were differentially expressed in the *rpoH1 *mutant at pH 7.0, in comparison to the wild type.

### Growth characteristics of *S. meliloti *wild type and *rpoH1 *mutant in response to an acidic pH shift

**Figure 3:**
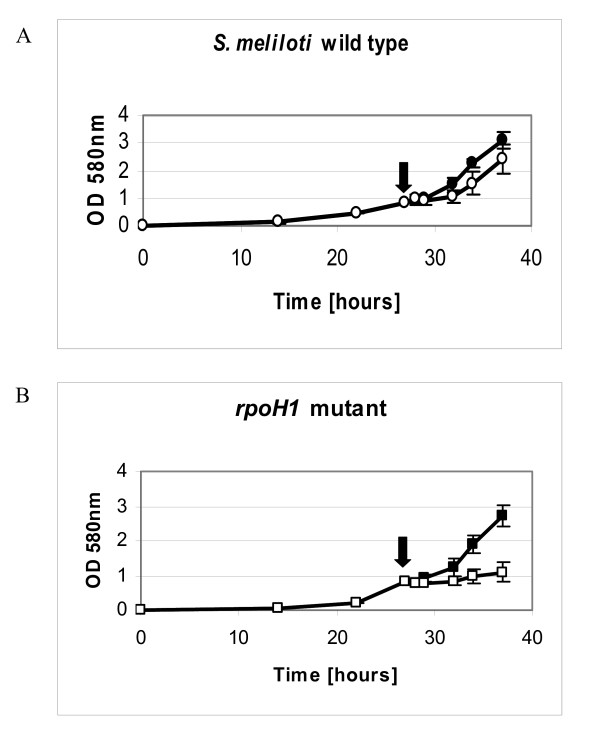
**Growth curves of *S. meliloti *1021 wild type strain and sigma factor *rpoH1 *mutant after pH shock. *S. meliloti *1021 wild type strain (A) and sigma factor *rpoH1 *mutant (B) were grown in medium at pH 7.0 and transferred to medium at pH 5.75 (open signs) or at pH 7.0 (filled signs)**. The arrows indicate the moment of pH shift. Cell growth was measured every two hours after pH shift. The error bars indicate the standard deviation calculated from three independent cultures.

Since the *rpoH1 *mutant is unable to grow at acidic pH, the RpoH1-dependent gene expression was investigated with a pH shift experiment. To this end, a growth test was performed in which *S. meliloti *wild type and *rpoH1 *mutant were transferred from neutral to acidic pH. This test was useful to determine if the *rpoH1 *mutant growth impairment was extended to sudden acidic pH shift and also to test further for a role for *rpoH1 *in pH shock response. *S. meliloti *wild type strain 1021 and the *rpoH1 *mutant were grown under identical conditions at pH 7.0 until an optical density of 0.8 at 580 nanometers was reached. The cultures were then centrifuged and resuspended in fresh medium either at pH 5.75 or at pH 7.0 (control). The samples continued to be measured for optical density, at two-hour intervals, after pH shift. The growth behavior of the *rpoH1 *mutant was similar to that of the wild type when the cells were transferred to medium at pH 7.0, whereas a growth deficiency was observed for the *rpoH1 *mutant in comparison to the wild type when the cells were transferred to medium at pH 5.75 (Figure 3), suggesting once more the participation of the RpoH1 sigma factor in fighting pH stress. We tested the viability of the mutant cells 30 minutes after pH shift by observing their ability to form colonies in TY plates incubated at 30°C overnight. The results indicated that the transfer to medium at acidic pH is not lethal to the *rpoH1 *mutant and the colony-forming ability of the mutant cultures is less than 20% lower than that of wild type cells (data not shown).

### Effects of an acidic pH shift on *S. meliloti *wild type and *rpoH1 *mutant assessed by time-course transcriptome analysis

In order to characterize the regulation of *S. meliloti *response to pH stress, the progressive transcriptomic response of both *S. meliloti *wild type and the *rpoH1 *mutant to sudden environmental acid shift was investigated by global gene expression time-course analyses. The experimental setup for the procedure with the wild type was identical to that of the *rpoH1 *mutant, allowing therefore for significant data comparison. With the aim to identify *S. meliloti *genes involved in pH stress, cells were grown in medium at pH 7.0 until reaching an optical density of 0.8 at 580 nm, and then transferred to medium at pH 5.75 or pH 7.0 (control). Cells were harvested at time points 0, 5, 10, 15, 30 and 60 minutes after the transfer. For each point of time, the microarray hybridization analyses were performed comparing the cells shocked at pH 5.75 with control cells again transferred to medium at pH 7.0. Log_2 _ratio or fold change of gene expression was obtained for each gene at each time point against the time-matched control and the normalized model-based expression values of genes were compared. In order to identify genes that play a role in the cellular response to acidic pH, significant change in expression was determined in combination with a cut-off value of approximately threefold change. That is, only genes that showed a significant increase or decrease in the expression ratio of circa threefold (M-value ≥ 1.4 or ≤ -1.4) between the two pH classes, for at least one of the six time points, were considered. Out of 14,000 array elements interrogated, a total of 210 nonredundant genes were selected, whose expression was altered significantly at one or more time points in the wild type arrays (Additional file 3).

Overall, the observed response of the *S. meliloti *wild type following acid shift is in agreement with that described by Hellweg *et al. *[30]. Most transcriptional changes occurred within 20 minutes after pH shift and upregulation was slightly dominant over downregulation at all time points. The response to acidic pH stress was characterized by an intricate variation in the expression of gene sets associated with various cellular functions over time. Among the most strongly upregulated genes (M-value ≥ 1.8) were *lpiA*, which codes for a low pH induced protein; *degP1*, which codes for the DegP1 serine protease; and *cah*, which codes for a carbonic anhydrase. Among the groups of genes responding to the shift to acidic pH were those of the exopolysaccharide I biosynthesis as well as flagellar and chemotaxis genes [34,35]. While the genes of the exopolysaccharide I biosynthesis were upregulated, the expression level of flagellar genes decreased in response to acidic pH. Other responding functional groups of genes were those coding for chaperone proteins, which were upregulated, and genes involved in nitrogen uptake and metabolism.

In the *S. meliloti rpoH1 *mutant arrays following acid shift, 132 of the 6,208 genes on the *S. meliloti *1021 microarray showed significant time-dependent variation in expression in at least one of the six time points. Those genes exhibited approximately threefold change in at least one time point throughout the 60 minute time-course. Approximately 30 annotated genes among the 132 genes that are differentially expressed in the *rpoH1 *mutant arrays are not found within the set of 210 genes that are differentially expressed in the wild type after pH shock. Among the genes most strongly induced in the *rpoH1 *mutant arrays were *nex18*, a gene that codes for a nutrient deprivation activated protein [37] and again *lpiA*. Both of these acid-induced genes display an extracellular stress response function [36]. Similarly to the wild type arrays, several genes of the flagellar regulon were repressed at low pH, whereas the genes of the exopolysaccharide I biosynthesis were upregulated. In contrast to the *S. meliloti *wild type, some genes coding for nitrogen uptake and metabolism and several genes coding for chaperone proteins were not observed among the differentially expressed genes in the *rpoH1 *mutant arrays (Additional file 4).

### Time-course microarray data of *S. meliloti *wild type following an acidic pH shift were grouped in 6 K-means clusters

**Figure 4:**
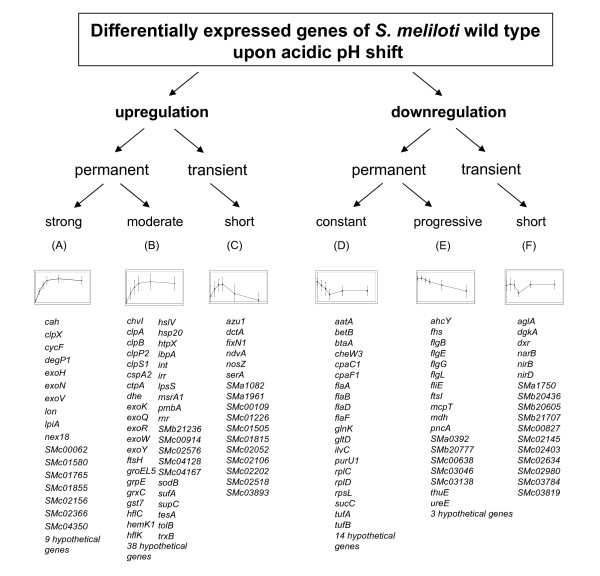
**K-means clustering of *S. meliloti *1021 genes differentially expressed after pH shift time-course**. Six clusters (A-F), calculated by K-means clustering, were characterized by their specific transcriptomic profiling over 60 minutes following acidic pH shift. The graphics illustrate the expression profile based on the mean values; the X-axis represents time, whereas the Y-axis represents the log_2 _ratio of gene expression (detailed view of the axes is shown in Figure 6). Tables below each graphic enlist genes distributed to the corresponding cluster.

In order to extract the fundamental patterns of gene expression from the data and to characterize the complex dynamics of differential expressions from a temporal viewpoint, clustering of genes that show similar time-course profiles was carried out. Genes with a significantly altered expression after pH shock were analyzed and clustering of the time-course data (log_2 _ratio of gene expression) was performed using the Genesis software [62], which is suited for analysis of short time-series microarray data. The K-means clustering method was implemented to define a set of distinct and representative models of expression profiles based on the mean values of similar expression data. With K-means, each gene groups into the model profile to which its time series most closely matches, based on its Euclidian distance to the profiles. Clustering analysis was performed on the 210 genes that displayed significant differential expression at one or more time points in the wild type arrays. Genes with similar expression characteristics were therefore grouped in the same cluster. A total of 6 clusters were generated for the wild type microarray data, with distinct expression patterns over the time-course. Clusters A to C represent the genes whose expression was upregulated and clusters D to F represent the genes whose expression was downregulated within the 60 minutes following pH shift (Figure 4, Additional file 5). Operons and genes involved in similar cellular functions were predominantly grouped in the same clusters.

Cluster A grouped genes with the strongest transcriptional induction after shift to low pH. It consists of 28 genes, including *nex18*, involved in the response to nutrient deprivation stress [37] and *lpiA*, involved in the formation of lysyl-phosphatidylglycerol, which is a low pH induced protein in *S. medicae *[38]. The exopolysaccharide biosynthesis genes *exoV*, *exoH*, *exoN*, and the gene for the Lon protease, a regulator of exopolysaccharide synthesis that is required for nodulation with alfalfa [39], also grouped in this cluster. Cluster B comprises genes that were gradually upregulated during the time-course and reached a plateau at approximately 20 minutes after pH shift. The genes in cluster B had, in comparison to the genes in cluster A, average lower M-values throughout the time course. This group includes several genes involved in exopolysaccharide I biosynthesis. The upregulation of exopolysaccharide biosynthesis genes upon sudden pH shift probably accounts for the mucoid phenotype in *S. meliloti *cells grown on plates at low pH and is in accordance to what has already been reported by Hellweg *et al.* (2008). Moreover, this cluster also includes a broad range of genes coding for heat shock proteins and chaperones involved in stress response, such as *ibpA*, *grpE*, *hslVU *and *groEL5 *and the genes coding for the proteases HflCK, HtpX, FtsH, ClpAB, ClpP1 and ClpS. Cluster C is composed of genes which were transiently induced after pH shift. It contains the dicarboxylate transport system DctA, which is essential for symbiosis in *S. meliloti *[40]. Also, the gene smc01505, which plays the function of the *anti*-sigma factor for the extracytoplasmic function sigma factor RpoE2 [41], was transiently upregulated (Figure 4).

Most genes in cluster D were gradually downregulated up to 30 minutes after pH shift, and maintained the peak of downregulation at 60 minutes. This cluster comprises a number of genes related to flagella biosynthesis and pillus assembly. Cluster E is composed of genes whose expression decreased continuously for the whole duration of the time-series experiment. The expression was gradually downregulated as of 5 minutes after pH shift, followed by greater downregulation up to 60 minutes. Among the genes in this cluster were the flagellar genes *flgG flgL*, *flgB *and *fliE*. Cluster F consists of genes which were transiently downregulated in their expression level after pH shift. It involves genes that play a role in nitrate assimilation, such as *nirB*, *nirD *and *narB *and the nitrate transporter *smb20436 *(Figure 4, Additional file 5).

### Analysis of expression profiles of the *S. meliloti rpoH1 *mutant following an acidic pH shift in view of wild type results

In order to elucidate the role of RpoH1 in transcription dynamics during pH stress response, the time-course transcriptomic analyses of the *rpoH1 *mutant upon acidic pH shift were compared to those of the wild type. For a most effective comparative analysis, K-means clustering was performed for the 210 genes selected through the filtering of the wild type data, but this time the clustering was carried out with their log_2 _expression data in the *rpoH1 *mutant arrays. This approach enabled the identification of genes that, throughout the time-course, behaved in a similar fashion both in the *rpoH1 *mutant arrays and in the wild type, as well as the identification of genes that displayed no differential expression in the *rpoH1 *mutant arrays, even though they were differentially expressed, upon acidic pH shift, in the wild type.

**Figure 5:**
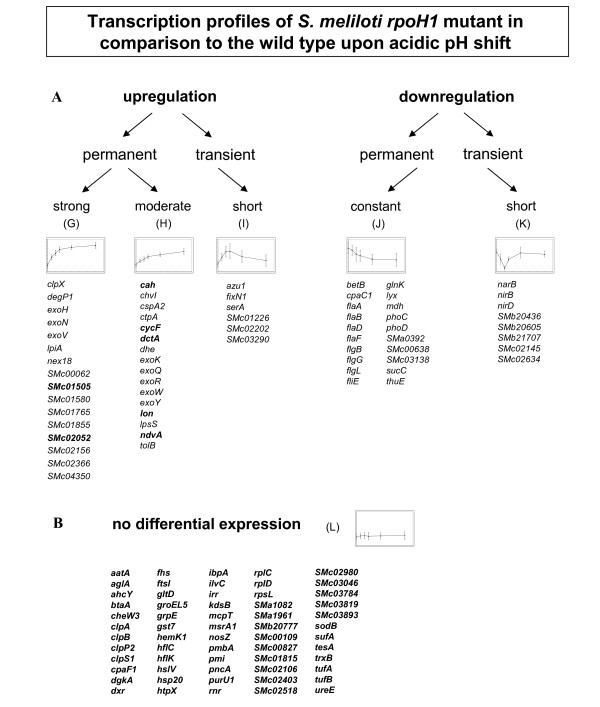
**Classification of expression profiles of *S. meliloti rpoH1 *mutant genes upon acidic pH shift in comparison to the wild type**. Representative genes are listed below graphics. Uniquely classified groups (G-L) were obtained through K-means clustering of *rpoH1 *mutant microarray data. The graphics illustrate the expression profile based on the mean values; the X-axis represents time, whereas the Y-axis represents the log_2 _ratio of gene expression (detailed view of the axes is shown in Figure 6). Genes marked in bold present dissimilar expression profile in comparison to *S. meliloti *wild type and therefore fit into a different cluster in the wild type clustering results.

The dynamic gene expression profiles were also catalogued into six clusters for the *rpoH1 *mutant, separating groups of genes with the highest possible similarity. Clusters G and H comprise genes that were constantly upregulated over time, either with a very strong induction (M-value ≥ 2.5 for at least one time point) or a moderate one (M-value ≤ 2.5) (Figure 5). Among the strongly upregulated genes in cluster G were *nex18 *and *lpiA*, the exopolysaccharide biosynthesis genes *exoV*, *exoH*, *exoN *and the gene coding for the Cah carbonic anhydrase, which is also induced in response to phosphate starvation of *S. meliloti *[42]. Genes grouped in cluster H include many *exo *genes and the gene coding for a regulator of succynoglycan production *chvI *[43], as well as the gene encoding the translocation protein TolB. A few transiently upregulated genes were listed in cluster I, such as the gene coding for SerA dehydrogenase (Figure 5A).

Clusters J and K grouped genes that were downregulated throughout the time-course, with persistent and transient downregulation, respectively. Like in the wild type arrays, many flagellar genes were also downregulated in the mutant and grouped in cluster J. The phosphate transport system encoded in the *phoCDET *operon also grouped in this cluster. In *E. coli*, *phoB *is involved in the acid shock response [44]. Among the transiently downregulated genes in cluster K were genes involved in nitrogen metabolism, such as those coding for nitrite and nitrate reductases, *nirD*, *nirB *and *narB*, which play a role in the conversion of nitrate to ammonia. Unlike the wild type, the clustering of the *rpoH1 *mutant data yielded the observation of a large cluster of genes whose expression changed very little throughout the time-course. For the genes in cluster L, the M-values remained close to zero at all time points (Figure 5B). Genes in cluster L include those coding for heat shock proteins and proteases, as well as the elongation factor *tufAB *operon and the gene coding for the putative chemotaxis protein *cheW3*. The complete lists of genes obtained from the clustering of the *rpoH1 *mutant data can be seen in Additional file 6. Additionally, in order to confirm the microarray results, quantitative reverse transcription PCR (qRT-PCR) analyses of six different genes were performed, for time points 10 and 60 minutes after pH shock (Additional file 7). The qRT-PCR results were very similar to those of the microarray expression data, for all genes analyzed, with the exception of the *dctA *gene, which presented a relatively higher expression value than that observed in the wild type microarrays at the 60-minute time point.

### Identification of *S. meliloti *genes that are regulated in an RpoH1-independent manner following an acidic pH shift

**Figure 6:**
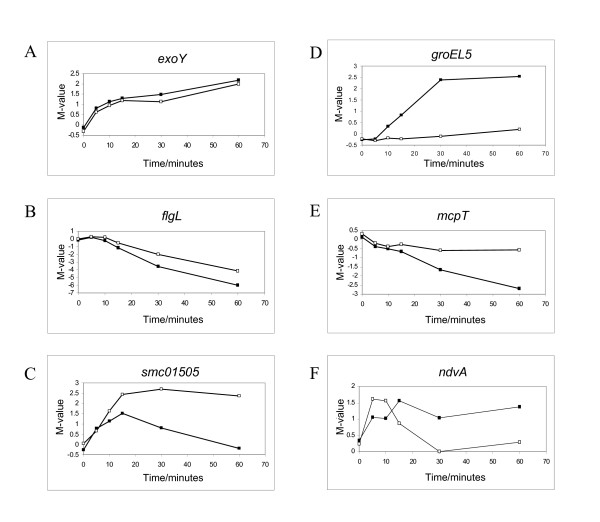
**M-values of specific genes throughout the time-course following acidic pH shift in *S. meliloti *1021 wild type strain (closed squares) and sigma factor *rpoH1 *mutant (open squares)**. Graphics A and B exemplify RpoH1-independent up and downregulation, respectively, whereas graphics D and E show RpoH1-dependently regulated genes. C and F account for complex RpoH1-dependent downregulation in the later time points following acidic shift.

Based on the cluster comparison between wild type and *rpoH1 *mutant, our results were most consistent with the dynamic distribution of genes in two different categories: genes whose expression at low pH is independent of *rpoH1 *expression and genes that display an expression dependent on *rpoH1 *after pH shift. RpoH1-independent genes were designated as those distributed into similar expression profiles in both wild type and *rpoH1 *mutant clustering analyses, that is, genes that were similarly up- or downregulated in both mutant and wild type arrays. Most genes from wild type cluster A presented an RpoH1-independent expression, as they were also upregulated in the *rpoH1 *mutant arrays and grouped at cluster G in the *rpoH1 *mutant clustering analysis. The gene coding for the low pH induced protein LpiA also presented RpoH1-independent upregulation in the pH shift arrays, as did the exopolysaccharide I biosynthesis genes *exoQ*, *exoW*, *exoV*, *exoH*, *exoK exoR*, *exoN*, and *exoY* (Figure 6A). Similar expression profiles could also be observed for the genes coding for the carbonic anhydrase Cah and the cytochrome CycF protein. Almost all genes involved in motility and flagellar biosynthesis, like the flagellar genes *flgB*, *fliE*, *flgG *and *flgL *(Figure 6B), displayed similar expression profiles in both wild type and mutant arrays, characterizing therefore a likely RpoH1-independent downregulation of motility genes upon acid pH shift in *S. meliloti*. Flagellar genes *flaA*, *flaB*, *flaD*, and *flaF *were also downregulated in the mutant, showing therefore that the absence of RpoH1 probably did not interfere with the reduction of cell motility at low pH.

### Identification of *S. meliloti *genes that are regulated in an RpoH1-dependent manner following an acidic pH shift

Genes classified as RpoH1-dependent did not present significant differential expression after pH shift in the *rpoH1 *mutant arrays, having shown otherwise a threefold differential expression for at least one time point in the wild type arrays. They comprise as many as 101 genes of the *S. meliloti *genome whose transcription after pH shift seems to be dependent on *rpoH1 *expression (Additional file 4). A number of protein turnover and chaperone genes were upregulated in the wild type arrays, such as the ones coding for the heat shock proteins IbpA, GrpE and GroEL5 (Figure 6D), as the ones coding for the Clp proteases, which are involved in the degradation of misfolded proteins [25]. No differential expression whatsoever was observed for those genes in the *rpoH1 *mutant arrays, characterizing thus an RpoH1-dependent expression of stress-response genes upon acid pH shift (Figure 5B, Additional file 6). Genes involved in translation, like *tufA *and *tufB, rplC rplD *and *rplS*, were downregulated, characterizing a seemingly RpoH1-dependent inhibition of translational activity in *S. meliloti *cells under pH stress. Genes *cheW*3 and *mcpT *(Figure 6E), coding for proteins involved in chemotaxis, were also downregulated only in the wild type arrays.

### Identification of *S. meliloti *genes that are regulated in a complex manner following an acidic pH shift

RpoH1 is also involved in the downregulation of specific transiently expressed genes. Interestingly, three genes from wild type cluster C were not grouped in cluster I as transiently upregulated in the *rpoH1 *mutant arrays. Those are the genes *dctA*, coding for a dicarboxylate transport protein, *ndvA*, coding for a beta glucan export protein, and the gene *smc01505*, which codes for the RpoE2 anti-sigma factor. These genes seem to have an RpoH1-independent upregulation, but an RpoH1-dependent downregulation as of 20 minutes following pH shift. In the wild type arrays, their expression is transient, but in the rpoH1 mutant arrays they remained upregulated throughout the entire time period analyzed (Figure 6C, F). For those genes, the RpoH1 sigma factor is likely to play a role in a more complex regulatory system with the involvement of a secondary regulator, for instance a repressor, which would be under the control of RpoH1, whereas the transcription activation of the genes themselves seems to be RpoH1-independent.

### Functional classification of genes regulated in an RpoH1-dependent manner

**Figure 7:**
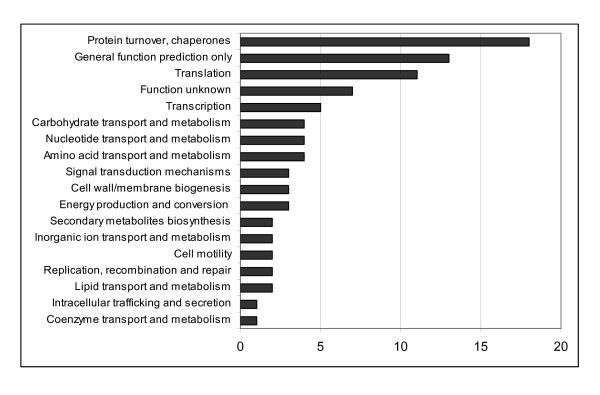
**COG classification of *S. meliloti *genes that are regulated in an RpoH1-dependent manner after shift to low pH**. The scaling of the X-axis indicates the number of genes assigned to each COG category.

The 101 genes that had distinct expression profiles in the *rpoH1 *mutant arrays in comparison to the wild type, ergo genes that presented an RpoH1-dependent expression, were also grouped according to their COG classification. The COG classification distributes genes in orthologous groups on basis of functional predictions and patterns of sequence similarities [45]. The RpoH1-dependent genes were assigned to 18 functional categories, indicating a global effect on gene expression dependent on RpoH1 upon pH shock. Among the known most representative classes were protein turnover and chaperones, followed by translation, transcription and by transport and metabolism of carbohydrates, nucleotides and amino acids (Figure 7). There is indeed a dramatic increase in the expression of chaperone proteins and heat shock genes in response to pH shock. A total of 24 genes that presented an RpoH1-dependent upregulation following acid shift are involved in heat shock and stress response. Among the proteases, the genes coding for HtpX, a membrane-bound and stress-controlled protease well characterized in *E. coli *[46], as well as those coding for ClpB and ClpP2, responsible for disassembling protein aggregates that accumulate in the cytoplasm under stress conditions [25], were expressed in dependence of RpoH1. The operon formed by the genes *hslUV*, which codes for an intrinsic ATP-dependent proteasome system for degradation of misfolded proteins in the cytoplasm, was also upregulated in an RpoH1-dependent fashion. Among the induced chaperones were also the gene *Smc00699*, coding for a heat shock DnaJ-like protein, as well as the gene coding for GrpE, which is part of the cellular chaperone machinery capable of repairing heat-induced protein damage [47]. Moreover, there was an RpoH1-dependent upregulation of the operon that codes for the only GroELS proteins specialized in stress response in *S. meliloti*, GroELS5 [25]. The gene coding for the small heat shock protein IbpA [48] was also upregulated. Genes like *groEL5 *and *clpB *have already been described as genes whose transcription is RpoH1-dependent in *S. meliloti *[22,25]. The group of proteins shown to be involved in the heat shock response under the transcriptional control of RpoH usually includes chaperones, proteases, and regulatory factors [49]. The mutation in the *rpoH1 *gene in *S. meliloti *and its characterization under pH stress revealed indeed a lack of activation of all major types of regulatory chaperones and key heat shock proteins usually activated in stress conditions. In the present study, we have seen representatives of all of those groups to be involved in pH stress response. We hence attest to the role of *rpoH1 *in *S. meliloti *pH stress response as being evidenced by the activation of acid-induced heat shock proteins and chaperones in dependence of *rpoH1 *expression.

## Discussion

### The *S. meliloti *sigma factor RpoH1 is important for stress response at low pH

In the soil, *S. meliloti *deals with adverse environmental variations that could induce physiological stress responses. Alternative sigma factors, such as RpoH1, directly sense and respond with transcriptional activation to the presence of stress conditions in their environment. The relative lack of differential expression of genes at pH 7.0 most likely reflects the absence of an inhospitable environmental condition to activate the alternative *rpoH1 *transcriptional response. The differential expression of genes related to rhizobactin synthesis in the microarray analyses may indicate a need for increased iron uptake regulation at pH 7.0. Even though the *rpoH1 *mutation does not affect host invasion during the endosymbiotic process, *rpoH1 *mutant bacteroids are defective in nitrogen fixation (Fix-- phenotype) [23]. However, we cannot explain the requirements for RpoH1 during symbiosis as a consequence of rhizobactin necessity, since rhizobactin is not expressed in the nodules [32].

The growth of the *rpoH1 *mutant was severely compromised at pH 5.75 and a growth defect was also observed after pH shock experiments. Growth inhibition probably occurs as a result of both lower internal pH and the differential ability of anions to inhibit metabolism. The fact that an *rpoH1 *mutant does not grow on LB plates containing acid pH gradient [25] corroborates our pH sensitivity phenotype. Previous studies have shown that an *rpoH1 *mutant is capable of eliciting the formation of nodules on alfalfa plants, but the *rpoH1 *mutation causes early senescence of bacteroids during the endosymbiotic process [23,25]. The present work did not explore regulation within the nodule, another condition in which *rpoH1 *is expressed [23]. Bearing in mind that the endosymbiotic process is affected by the ability of rhizobial cells to protect themselves against environmental stresses encountered within the host, it is possible that the early senescence observed for *rpoH1 *mutant nodules [25] is caused by an increased sensitivity to pH stress upon rhizosphere and plant acidification during nodulation. Within the plant cell, symbiotic bacteria have to face acid conditions [50]. Transport of protons or ionized acids could acidify the symbiosomes and the low oxygen concentration in the nodules could be expected to alter pathways of carbon metabolism, leading to the production of organic acids that inhibit the regulation of cytoplasmic pH [50]. In this case the role of RpoH1 during pH shift would be paramount not only at free-living growth, as shown in this work, but also during symbiosis, and sensitivity to low pH values is very likely the reason *rpoH1 *mutant cells cannot form functional nodules. Several stress responses are known to be linked with pH stress, including oxidative stress, heat shock, and envelope stress. Low pH usually accelerates acid consumption and proton export [51], and increases production of oxygen radicals, thus inducing a partial oxidative stress response. In this work, the expression of genes coding for ATP transporters that can work as proton pumps and proteins involved in osmotic stress response seem to be at least partially dependent on RpoH1. Likewise, the RpoH sigma factor has already been implicated in the oxidative stress response in other rhizobia [9,11].

Moreover, our study revealed patterns of pH response and clarified the overlap of pH stress with heat shock response. The heat shock response in bacteria is characterized by the induction of a number of proteins in response to change in temperature. Since many of these proteins are also induced by a variety of other environmental stress conditions, it can be concluded that such response is a stress response and not only a heat shock response. RpoH1 has been described in *S. meliloti *as the heat shock response sigma factor [23-25]. The group of proteins shown to be involved in the heat shock response under the transcriptional control of RpoH1 includes chaperones, proteases, and regulatory factors. In the present study, we have seen that those groups of proteins are also involved in pH stress response. Hence, the pH stress response in *S. meliloti*, characterized in this work, is likewise not specific for pH stress, but also likely to be a response to other types of environmental stress.

### Three groups of *S. meliloti *genes were found to be transcriptionally regulated upon pH stress in an RpoH1-independent, in an RpoH1-dependent and in a complex manner

Overall, gene expression following rapid acid shift revealed several patterns of acid stress response, characterized by the induction of heat shock regulons and exopolysaccharide production and the repression of energy-expensive flagellar and chemotaxis regulons. The observed response of the *S. meliloti *wild type following acid shift is in agreement with that described by Hellweg *et al. *[30]. Though the nomenclature adopted in this manuscript is similar to that found in Hellweg *et al*., cluster distribution differs in that Hellweg divided the dataset in eight clusters and in the present study the dataset was divided into six clusters. Three classes of transcriptionally regulated *S. meliloti *genes could be identified: genes which were regulated in an RpoH1-independent, an RpoH1-dependent or in a complex manner upon pH stress. The first class of genes, which were regulated in an RpoH1-independent manner, comprises exopolysaccharide I biosynthesis genes, like *exoQ, exoP, exoN *and *exoY*, and also the group of genes involved in motility and flagellar biosynthesis like the flagellar genes *flgA, flgL *and *mcpT *[35]. Those expression patterns further confirm the notion of an induced exopolysaccharide production and a hampered motility activity of *S. meliloti *upon pH shock [30], though the induction of exopolysaccharide production and the repression of motility is most seemingly an RpoH1-independent process. The vast majority of chemotaxis and flagellar genes was indeed downregulated in a similar fashion in both wild type and mutant arrays, even though the chemotaxis gene *cheW3*, for instance, was not repressed in the *rpoH1 *mutant. The genes included in this class of RpoH1-independently regulated genes do not, as a rule, comprise genes with a specific stress response function.

The second class of *S. meliloti *genes, which comprises those genes that responded in an RpoH1-dependent manner, is composed of genes known to be involved in heat shock, such as *ibpA, grpE, clbP *and *groEL5*, as well as some genes involved in translation like *tufA *and *rplC*. Our analysis strongly suggests that a transcriptional response to pH takes place in which cells reallocate resources by inhibiting energy-consuming processes and upregulating transcription of genes involved in chaperone mechanisms. The heat shock regulons were clearly under the control of RpoH1, and though genes belonging to diverse functional classes were transcriptionally modulated by *rpoH1 *expression, the most represented class of genes induced by pH shock stress was by far that of genes coding for chaperones. Those genes are likely to be paramount for an appropriate cellular response in fighting pH stress. The finding of genes coding for chaperone proteins such as *groESL5 *and *clpB*, already known to be RpoH1-dependent after temperature upshift [25] remarkably attests to the reliability of our results. The *groEL5 *mutant is able to fix nitrogen in the nodules [25]. However, other important pH stress response genes such as *lon*, *grpE *and *ibpA *[39,47,48] are under the control of *rpoH1 *in *S. meliloti *and could be involved in dealing with the low pH environment in free-living conditions and within the nodule.

The third class was that of genes regulated in a complex manner. This was the case for the genes *ndvA *and *smc01505*, which were transiently upregulated only in the wild type arrays, whereas in the *rpoH1 *mutant arrays those genes were constantly upregulated. This lack of downregulation implies most likely that a secondary regulation takes place, in which a repression of the activities of some genes is then dependent on *rpoH1 *expression. Interestingly, *smc01505 *codes for the RpoE2 anti-sigma factor. RpoE2 is known to be involved in general stress response and in oxidative stress response in *S. meliloti *[41,52], though it has been suggested that RpoE2 is not necessary for stress adaptation [52]. Gene expression patterns are also influenced by sigma factor availability and activity. In the time-course comparison, *smc01505 *was regulated differently from the wild type in the *rpoH1 *mutant. It is likely that in a complex event such as stress response, extracytoplasmic sigma factors like RpoE2 act together with RpoH1, which is a sigma factor mostly responsible for stress response in the intracellular compartment. In *E. coli*, for instance, *grpE *expression is under the regulation of the sigma 70 and sigma 32 [47] and *rpoH *transcription is controlled by sigma 70, sigma E and sigma 54 [53]. Many stress genes are also regulated by transcriptional repressors and activators, a number of which were induced at the transcription level in our experiments. Those constitute a secondary activation and are important for responding to specific intracellular cues and for precisely coordinating transcription changes with the physiological state of the cell. Therefore, in order to understand how stress response in the periplasm and cytoplasm are coordinated, it is necessary to dissect the transcriptional regulatory network of sigma factors, considering not only that secondary regulation and cross-regulation take place, but also that there can be binding sites for more than one sigma factor in the promoter region of genes involved in stress response. Our primary focus with the time-course microarray analyses was to identify genes that are part of the regular pH stress response in *S. meliloti *wild type and from there to pinpoint genes whose expression is dependent on *rpoH1 *expression. This approach facilitated the comparison, for the genes that were differentially expressed only in the *rpoH1 *mutant arrays are probably under the control of more complex genetic circuits and require more extensive analyses for their role in stress response to be elucidated. Moreover, successful validation of the microarray data was obtained by qRT-PCR analyses performed for six different genes that were differentially expressed in the wild type. In the group of genes analyzed, RpoH1-dependent, RpoH1-independent and complex regulation could be observed, in accordance to the microarray expression data. The only dissimilarity in the qRT-PCR results was observed for the *dctA *gene, whose results were inconclusive for the wild type at the 60-minute time point. It may be that the upregulation of the *dctA *gene is sustained throughout the time-course. On the other hand, the available qRT-PCR data do not admit predictions about expression values between 10 and 60 minutes. Although the M-values were generally higher in the qRT-PCR analyses, the genes showed very similar expression patterns to those observed in the microarrays, indicating that the results can indeed be trusted (Additional file 7).

### Time-course global gene expression is a powerful tool for the identification of *S. meliloti *genes regulated by the sigma factor RpoH1

The RpoH1-dependent pH stress response of *S. meliloti *was characterized with the aid of transcriptomic studies. Microarray hybridization was therefore employed to investigate the time-course response of *S. meliloti *to a sudden acid shift. Time-course experiments of gene expression facilitate the understanding of the temporal structure of regulatory mechanisms and the identification of gene networks involved in stress response [54]. The time-series microarrays, followed by clustering, enabled us to capture multiple expression profiles at discrete time points of a continuous, but dynamic, cellular process. Also, it enabled us to extract the fundamental patterns of gene expression inherent in the data.

In *S. meliloti*, two RpoH-type sigma factors are annotated in the genome [21]. RpoH1 and RpoH2 are involved in different stress responses, and this probably provides increased capacity for *S. meliloti *to adapt to different environments. We suggest for the first time that RpoH1 efficiently regulates the expression of specific heat shock genes in response to pH stress in *S. meliloti*. This type of regulation structure would also be efficient for adjustment to other stresses requiring rapid change of metabolic mode as well as thermal adaptation. We ultimately conclude that RpoH1 is necessary for the dynamic response of *S. meliloti *to sudden pH shift and it accounts for critical changes in gene expression during pH stress response. These findings form a basis for subsequent analyses of regulation and function of the stress response in *S. meliloti*. The time-course study provides efficient methodology for hypothesis-driven investigations to dissect the roles of sigma factors and other key players in transcription regulation not only in pH stress conditions, but in general stress response and adaptation. In addition to the recognition of individual genes with altered expressions, the proposed method for clustering of time-course data enabled us to identify gene clusters, each with a unique time-dependent expression pattern. Further biochemical and genetic studies on the regulatory events of *S. meliloti *cells undergoing environmental stress should continue to provide useful information for further understanding of the role of RpoH1 and other alternative sigma factors in stress response.

## Conclusions

Our study indicated that sigma factor RpoH1 plays an important role in the response to low pH stress in *S. meliloti*. This role was efficiently unraveled by time-course microarray studies, in which key players involved in stress response whose transcription is under regulation of RpoH1 were identified. Clustering of time-course microarray data of *S. meliloti *wild type and *rpoH1 *mutant allowed for the classification of three groups of genes that were transcriptionally regulated upon pH stress in an RpoH1-independent, in an RpoH1-dependent or in a complex manner. Among the genes that showed an RpoH1-dependent regulation, there were several coding for heat shock and chaperone proteins. Time-course global gene expression analyses can be further employed to facilitate the temporal study of regulatory mechanisms and provide a more comprehensive framework for studying dynamic cellular processes, such as stress response.

## Methods

### Bacterial strains, plasmids, and growth conditions

The bacterial strains and plasmids used in this work are listed in Table 1. *E. coli *strains were grown at 37°C in Luria-Bertani medium [55]. *S. meliloti *strains were grown at 30°C in tryptone yeast extract (TY) complex medium [56] or Vincent minimal medium (VMM) [57]. When required, antibiotics were supplemented to the media at the following concentrations: neomycin, 100 μg/ml; kanamycin, 50 μg/ml; and streptomycin, 600 μg/ml. The pH of the VMM was adjusted by using either HCl or NaOH.

**Table 1:** Bacterial strains, plasmids and PCR primers used in this study

	**Characteristics**	**Reference**
*Sinorhizobium meliloti*		
Rm 1021	Spontaneous mutant of wild type strain RU47, Sm^r^	[64]
Rm 1021Δ*rpoE1*	Rm1021 derivative, *rpoE1 *mutant	This study
Rm 1021Δ*rpoE2*	Rm1021 derivative, *rpoE2 *mutant	This study
Rm 1021Δ*rpoE5*	Rm1021 derivative, *rpoE5 *mutant	This study
Rm 1021Δ*rpoH1*	Rm1021 derivative, *rpoH1 *mutant	This study
Rm 1021Δ*fecI*	Rm1021 derivative, *fecI *mutant	This study
		
*Escherichia coli*		
DH5_MCR	F^- ^*endA1 supE44 thi-1 *λ^- ^*recA1 gyrA96 relA1 deoR *Δ(*lacZYA-argF*)*U169 *ϕ80d*lacZ*ΔM15 *mcrA *Δ(*mrr hsdRMS mcrBC*)	[65]
S17-1	*E. coli *294::[RP4-2(Tc::Mu)(Km::Tn*7*)] *pro res *Δ*recA *Tpr	[55]
		
Plasmids		
pK18mobsacB	pUC18 derivative, *sacB lacZ*α Km^r^, mobilizable	[58]
pJrpoH1	pJN105 derivative, *rpoH1*, Gm^r^	This study
		
*Primers*		
DEL_rpoE1_A	AGTAGGATCCGCGATCAGGAGGTCAT	This study
DEL_rpoE1_B	GTCCTTCATCGCTTCGGCAACCGGCATCAATTCCAG	This study
DEL_rpoE1_C	CTGGAATTGATGCCGGTTGCCGAAGCGATGAAGGAC	This study
DEL_rpoE1_D	AGTCGGATCCACGATCCTCTGCGTTGAAGC	This study
DEL_rpoE2_A	ATCGGAATTCGCTCGTCCTCGATGAT	This study
DEL_rpoE2_B	AACGAAGGCACGCGAGGTGACACGCTTGAACTCTTGG	This study
DEL_rpoE2_C	CCAAGAGTTCAAGCGTGTCACCTCGCGTGCCTTCGTT	This study
DEL_rpoE2_D	AGCGGAATTCAACCGCGACGGTTCCTATC	This study
DEL_rpoE5_A	GCGCAAGCTTCTGCAGGATGGAAGCGATT	This study
DEL_rpoE5_B	CTCGTCCGCTCAGTTCAATTGTCGCGATGCGTGACC	This study
DEL_rpoE5_C	GGTCACGCATCGCGACAATTGAACTGAGCGGACGAG	This study
DEL_rpoE5_D	ACGTAAGCTTGCCGACCAGAACCGTAA	This study
DEL_rpoH1_A	CGAAGACAGCGACGATGCAC	This study
DEL_rpoH1_B	ACCAGCCAATCCTGCCACTGCTCGAACTTCTTGACCGCCT	This study
DEL_rpoH1_C	AGGCGGTCAAGAAGTTCGAGCAGTGGCAGGATTGGCTGGT	This study
DEL_rpoH1_D	TATGAAGAGAGGCTCGGCCA	This study
DEL_fecI1_A	CGCGCATTGGTCGTGCGATT	This study
DEL_fecI1_B	GGTGCCGCAGGTACATGTGA	This study
DEL_fecI1_C	TCACATGTACCTGCGGCACCAGGCCTCGACCATGACGAAT	This study
DEL_fecI1_D	GATCGTGCGCCACATCGAAG	This study

### Construction of sigma factor mutants

The protocols of Sambrook *et al. *[55] were used for DNA manipulations. DNA fragments containing at least 500 base-pair deletions in the sigma factor genes were constructed by Gene Splicing by Overlap Extension or gene SOEing [31]. In general, most of the coding sequence of the genes was deleted, and only the nucleotides coding for the first and last two amino acids of the genes are still present in the mutant strains. In a first Polymerase chain reaction (PCR), regions up- and downstream of the desired deletion were amplified, and then they were fused in a second PCR. The primers used for this purpose are listed in Table 1. The deletion constructs obtained were subsequently cloned into the suicide vector pK18mobsacB, which allows sucrose selection for vector loss [58]. The resulting plasmids were conjugated into *S. meliloti *via *E. coli *S17-1 to introduce deletions by allelic exchange. Production of mutant strains was confirmed by PCR reactions designed to amplify DNA fragments spanning the gene of interest.

### CAS siderophore assay

Chrome azurol S (CAS) assay mixtures for siderophore detection were prepared as described by Schwyn and Neilands [33]. Supernatants of *S. meliloti *cultures grown in VMM were mixed 1:1 with a CAS assay solution. After equilibrium was reached, the absorbance at 630 nanometers was measured. The relative siderophore activity was determined by measuring optical density ratios of different cultures.

### Procedures for continuous pH and pH shift growth experiments

*S. meliloti *strains were grown in Vincent minimal medium (VMM) [57] at 30°C at either pH 7.0 or pH 5.75 for growth tests at continuous pH values. VMM medium was composed of 14.7 mM K_2_HPO_4_, 11.5 mM KH_2_PO_4_, 0.46 mM CaCl_2_, 0.037 mM FeCl_3_, 1 mM MgSO_4_, 15.7 mM NH_4_Cl, 10 mM sodium succinate, 4.1 μM biotin, 48.5 μM H_3_BO_3_, 10 μM MnSO_4_, 1 μM ZnSO_4_, 0.5 μM CuSO_4_, 0.27 μM CoCl_2_, and 0.5 μM NaMoO_4_. Triplicate samples were measured for optical density at 580 nm, twice a day, for 7 days. For pH shift experiments cells of three independent cultures were grown in 30 ml of VMM with pH 7.0 to an O.D._580 _of 0.8. Cell cultures of each flask were then centrifuged (10,000 × g, 2 min, 30°C) and the supernatant was discarded. The cell pellets were resuspended in 30 ml VMM with pH 5.75 or 30 ml VMM with pH 7.0 (control) and incubated at 30°C. At six time points cell suspension samples of 5 ml were harvested from each flask and immediately centrifuged (10000 × g, 1 min, 4°C). The resulting pellets were instantly frozen in liquid nitrogen for later RNA preparation. Cell suspension samples were harvested at 0, 5, 10, 15, 30, and 60 minutes following the pH shift. To determine the number of viable cells, dilutions of *S. meliloti *cultures grown 30 minutes after pH shift were plated on TY agar and incubated overnight at 30°C.

### RNA isolation

RNA was isolated according to the protocol published by Rüberg *et al. *[59]. Total RNA was prepared using the RNeasy mini kit (QIAGEN, Hildesheim, Germany). By ribolysation (30 s; speed, 6.5; Hybaid, Heidelberg, Germany) cells were disrupted in the RLT buffer provided with the kit in Fast Protein Tubes (Qbiogene, Carlsbad, CA).

### Transcriptional profiling using the SM14kOligo whole genome microarray

For microarray hybridization, three independent bacterial cultures from each condition were prepared as biological replicates for RNA isolation. Accordingly, for each time point, dual-fluorescence-labeled cDNA probes were prepared to hybridize with three slides, respectively. For each preparation of Cy3 and Cy5 labeled cDNAs, 10 μg of total RNA were used [60]. To each microarray, the cDNA of the pH 7.0 and pH 5.75 grown cultures were mixed and hybridized. Slide processing, sample hybridization, and scanning procedures were performed applying the Sm14kOligo microarray, that carries 50 mer to 70 mer oligonucleotide probes directed against coding regions and intergenic regions [61]. Analysis of microarray images was carried out applying the ImaGene 6.0 software (BioDiscovery) as described previously [42]. Lowess normalization and significance test (fdr) were performed with the EMMA software [60]. M-values (log_2 _experiment/control ratio), P-values (*t *test) and A-values were also calculated with EMMA. The M-value represents the logarithmic ratio between both channels. The A-value represents the logarithm of the combined intensities of both channels. The microarray results were verified for specific genes by quantitative reverse transcription-PCR using a QuantiTect SYBR Green reverse transcription-PCR kit (QIAGEN, Hildesheim, Germany) according to the manufacturer's instructions.

### Filtering and clustering analysis of the microarray data

K-means clustering analysis of the microarray time-course data was performed with the aid of the Genesis software [62]. After normalization, only genes with approximately threefold change in expression (M-value of ≥ 1.4 or ≤ -1.4) in at least one point of time in the wild type microarrays were considered for clustering analysis. Genes that did not present an evaluable expression value for at least 5 of the 6 points of time (missing values on the microarray flagged as empty spots) were not considered. K-means clustering was used for distributing differentially regulated genes into 6 groups, both with the wild type and with the *rpoH1 *mutant microarray data.

### Quantitative RT-PCR analyses

Reverse transcription was performed using Superscript II reverse transcriptase (Invitrogen) with random hexamers as primers. RNA samples were tested for two time points, 10 and 60 minutes after pH shock. Real-time PCRs were run on an Opticon system (BioRad) using the FastStart DNA MasterPLUS SYBRGreen I kit (Roche) according to the manufacturer's instructions. The housekeeping gene *rkpK *was used as a reference for normalization. The sequences of the primers used are available at http://www.cebitec.uni-bielefeld.de/groups/brf/software/gendb_info/. Three independent cultures were analyzed, as well as three technical replicates, for each time point.

### Microarray data accession numbers

The entire set of microarray data has been deposited in the ArrayLims database [63].

## Authors' contributions

DKCL carried out the molecular genetic studies, the statistical analysis and wrote the manuscript. SW and AP participated in the design of the study, revised it critically for important intellectual content and have given final approval of the version to be published.

## Supplementary Material

Additional file 1 **Complementation of *rpoH1 *mutation**. To verify if the complementation of the *rpoH1 *mutant phenotype could be achieved, a growth test was performed with *rpoH1 *mutant cells bearing a plasmid that contains the *rpoH1 *gene. Besides the *S. meliloti *wild type strain and the *rpoH1 *mutant bearing the recombinant plasmid, the wild type *S. meliloti *bearing the empty plasmid was also analyzed. All samples were grown in Vincent minimal medium and measured as triplicates, twice a day, for five days. As expected, the restoration of the wild type growth phenotype was observed for the *rpoH1 *mutant carrying the recombinant plasmid with the *rpoH1 *gene. Click here for file

Additional file 2 **CAS assay**. The CAS reagent provides a non-specific test for iron-binding compounds. The reaction rate established by color change is a direct indicator of the siderophore-concentration. CAS time-course test for assessment of siderophore production was performed with *rpoH1 *mutant and *S. meliloti *wild type by measuring the optical density of their CAS-assay supernatant at 630 nm for five minutes, in 15-second intervals. 630 nm is the wavelength for red and orange, colors that indicate presence of siderophores in the solution. Click here for file

Additional file 3 **Spreadsheet of *S. meliloti *wild type genes that were differentially expressed following acidic pH shift**. Spreadsheet of the 210 genes which were differentially expressed in *S. meliloti *wild type following acidic pH shift, with the name of each gene and its corresponding annotation, as well as the M-values calculated for each time point (0, 5, 10, 15, 30 and 60 minutes after pH shift) of the time-course experiment. Click here for file

Additional file 4 **Spreadsheet of *rpoH1 *mutant genes used for expression profiling following acidic pH shift**. Listed are the 210 genes used for analysis of *rpoH1 *mutant expression profiling following acidic pH shift, with the name of each gene and its corresponding annotation, as well as the M-values calculated for each time point (0, 5, 10, 15, 30 and 60 minutes after pH shift) of the time-course experiment. Click here for file

Additional file 5 **Heat maps of clusters A to F**. The transcriptional data obtained by microarray analysis of the *S. meliloti *1021 pH shock experiment were grouped into six K-means clusters (A-F). Each column of the heat map represents one time point of the time-course experiment, after shift from pH 7.0 to pH 5.75, in the following order: 0, 5, 10, 15, 30 and 60 minutes. The color intensity on the heat map correlates to the intensity (log ratio) of the expression of each gene at the specified time point, with red representing overexpression and green indicating reduced expression. Click here for file

Additional file 6 **Heat maps of clusters G to L**. The transcriptional data obtained by microarray analysis of the *S. meliloti rpoH1 *mutant following acidic pH shift was analyzed taking into consideration the 210 genes that were also analyzed in the wild type experiments. The *rpoH1 *mutant microarray data were also grouped into six K-means clusters (G-L). Each column of the heat map represents one time point after shift from pH 7.0 to pH 5.75 of the time-course experiment, in the following order: 0, 5, 10, 15, 30 and 60 minutes. The color intensity on the heat map correlates to the intensity (log ratio) of the expression, with red representing overexpression and green indicating reduced expression. Click here for file

Additional file 7 **Quantitative RT-PCR**. qRT-PCR was performed for validation of the microarray expression data. The six genes used in the experiment were *smb20611*, *smc01505*, *grpE*, *lpiA, exoY *and *mcpT*. Differences in gene expression were determined by comparing the crossing points of samples measured in three replicates. Comparison of expression data was always performed between samples transferred to medium at pH 5.75 and control samples transferred to control medium at pH 7.0, 10 or 60 minutes after pH shift. In the group of genes analyzed, RpoH1-dependent, RpoH1-independent and complex regulation could be observed, in accordance to the microarray expression data. Section A includes the results obtained by qRT-PCR. The M-values of the microarray were included in section B to facilitate the comparison. Click here for file
