# Supplementary material for: The role of sigma factor RpoH1 in the pH stress response of Sinorhizobium meliloti
Source: BMC Microbiol. 2010 Oct 18;10:265. doi: 10.1186/1471-2180-10-265 (PMC2976971; doi:10.1186/1471-2180-10-265)
Supplement: Additional file 1 — Complementation of rpoH1 mutation. To verify if the complementation of the rpoH1 mutant phenotype could be achieved, a growth test was performed with rpoH1 mutant cells bearing a plasmid that contains the rpoH1 gene. Besides the S. meliloti wild type strain and the rpoH1 mutant bearing the recombinant plasmid, the wild type S. meliloti bearing the empty plasmid was also analyzed. All samples were grown in Vincent minimal medium and measured as triplicates, twice a day, for five days. As expected, the restoration of the wild type growth phenotype was observed for the rpoH1 mutant carrying the recombinant plasmid with the rpoH1 gene. [file 1471-2180-10-265-S1.PDF]

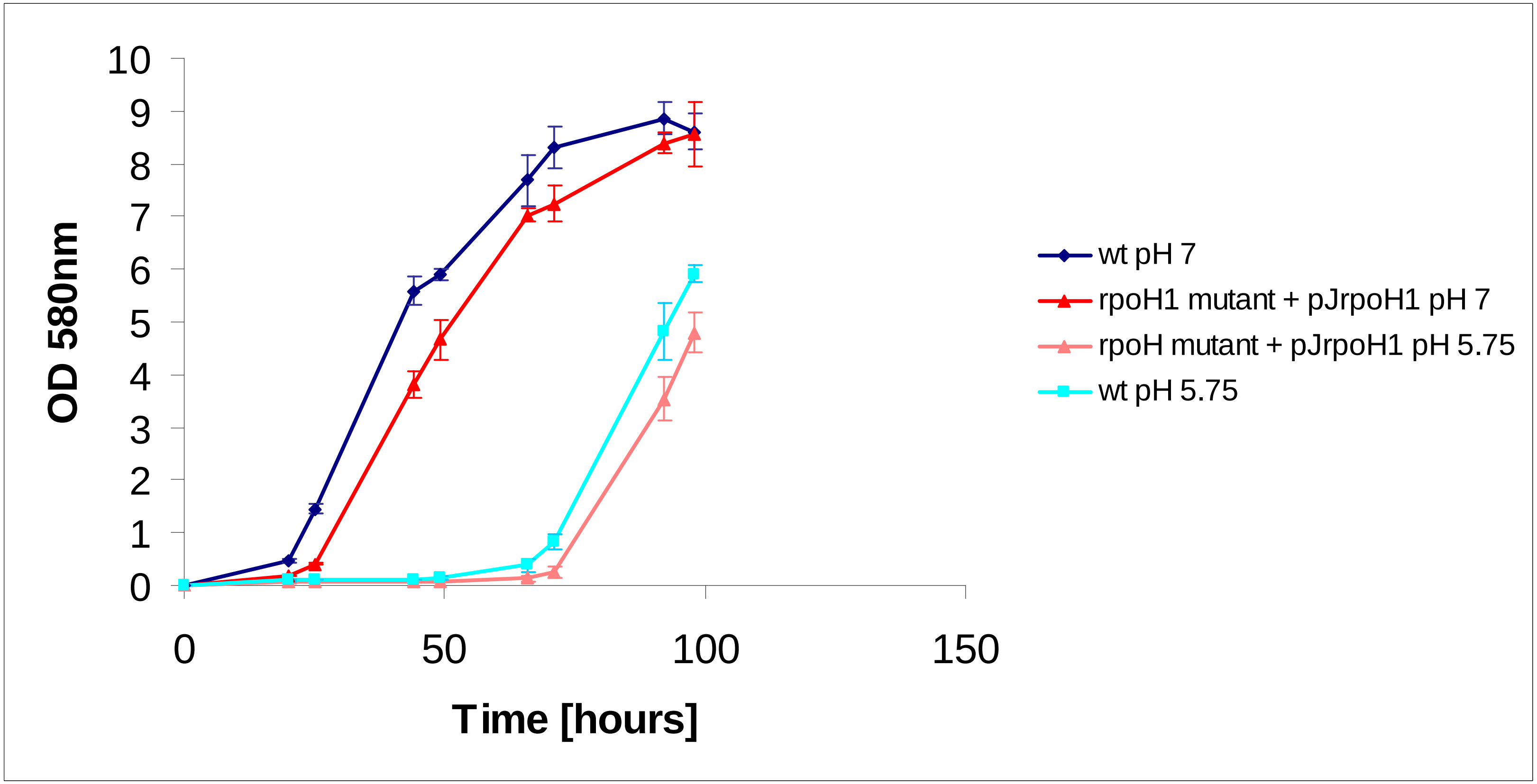

Additional File 1. Complementation of *rpoH1* mutation. Growth cultures of the *S. meliloti* wild type strain and the *rpoH1* mutant carrying the plasmid pJrpoH1 containing the *rpoH1* gene were performed at both pH 7 and pH 5.75. Strains were grown in Vincent minimal medium and measured for optical density at 580 nm at different time points, for five days. The error bars indicate the standard deviation calculated from three independent cultures.
