# Supplementary material for: The role of sigma factor RpoH1 in the pH stress response of Sinorhizobium meliloti
Source: BMC Microbiol. 2010 Oct 18;10:265. doi: 10.1186/1471-2180-10-265 (PMC2976971; doi:10.1186/1471-2180-10-265)
Supplement: Additional file 2 — CAS assay. The CAS reagent provides a non-specific test for iron-binding compounds. The reaction rate established by color change is a direct indicator of the siderophore-concentration. CAS time-course test for assessment of siderophore production was performed with rpoH1 mutant and S. meliloti wild type by measuring the optical density of their CAS-assay supernatant at 630 nm for five minutes, in 15-second intervals. 630 nm is the wavelength for red and orange, colors that indicate presence of siderophores in the solution. [file 1471-2180-10-265-S2.PDF]

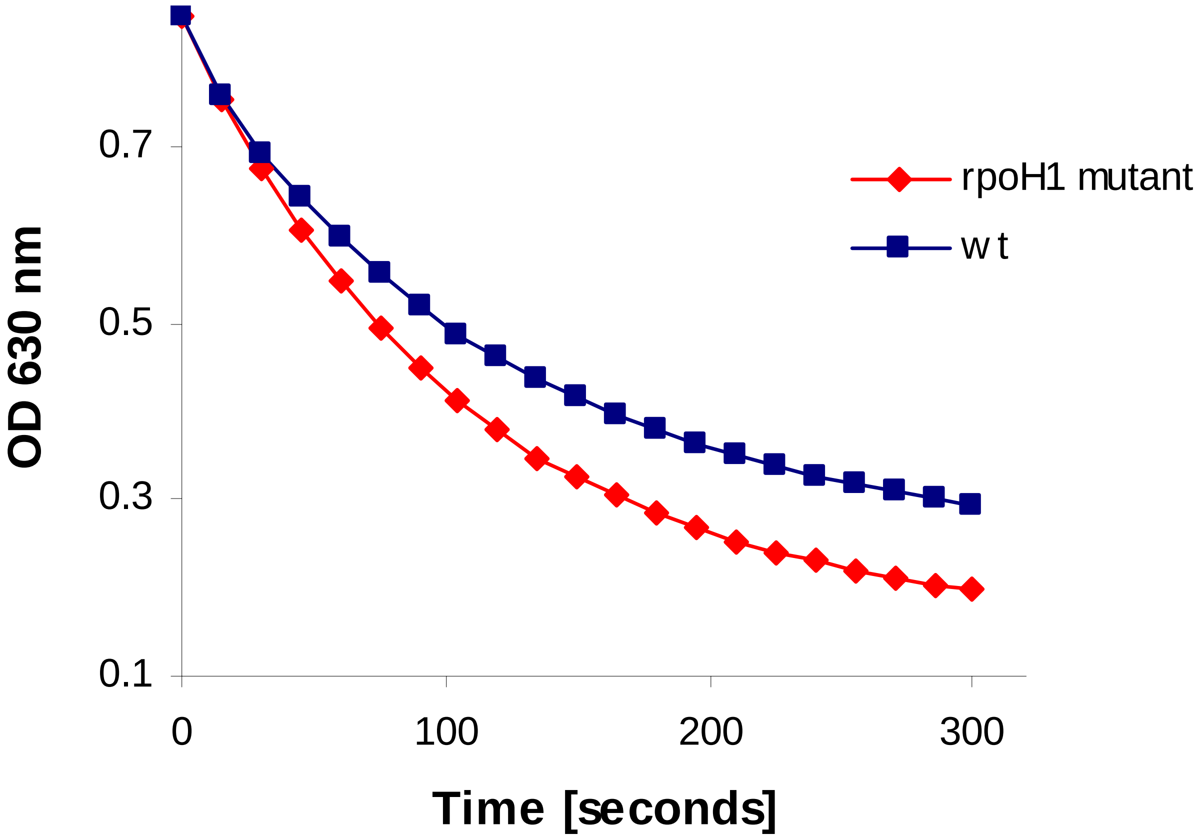

Additional File 2. CAS test for assessment of siderophore production. The formation of the iron-siderophore complex results in a color change from blue to orange when the CAS dye is released The time-course experiment was performed by measuring the optical density of the CAS-assay supernatant at 630nm for five minutes, in 15-second intervals.
