# Supplementary material for: The role of sigma factor RpoH1 in the pH stress response of Sinorhizobium meliloti
Source: BMC Microbiol. 2010 Oct 18;10:265. doi: 10.1186/1471-2180-10-265 (PMC2976971; doi:10.1186/1471-2180-10-265)
Supplement: Additional file 6 — Heat maps of clusters G to L. The transcriptional data obtained by microarray analysis of the S. meliloti rpoH1 mutant following acidic pH shift was analyzed taking into consideration the 210 genes that were also analyzed in the wild type experiments. The rpoH1 mutant microarray data were also grouped into six K-means clusters (G-L). Each column of the heat map represents one time point after shift from pH 7.0 to pH 5.75 of the time-course experiment, in the following order: 0, 5, 10, 15, 30 and 60 minutes. The color intensity on the heat map correlates to the intensity (log ratio) of the expression, with red representing overexpression and green indicating reduced expression. [file 1471-2180-10-265-S6.PDF]

CLUSTER G

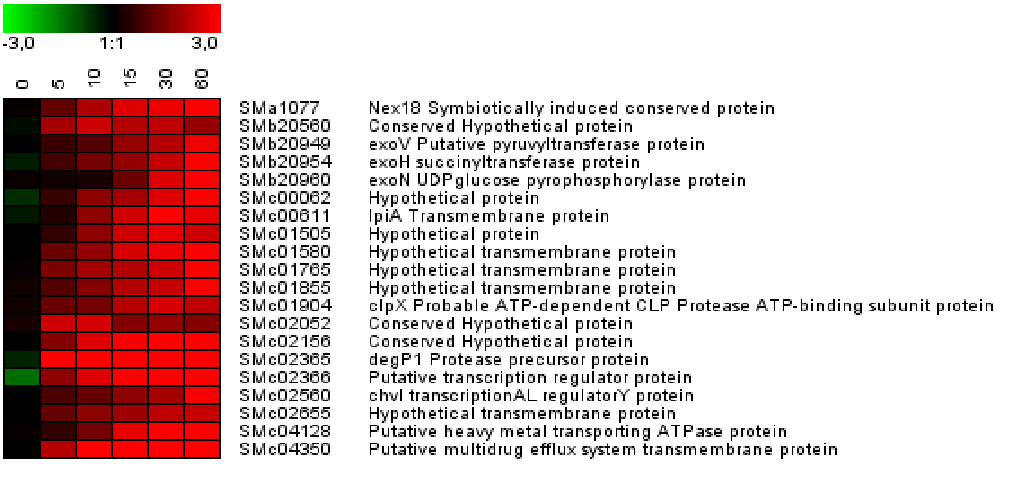
-3,0
1:1
3,0
0
5
10
15
30
60
SMa1077 Nex18 Symbiotically induced conserved protein
SMb20560 Conserved Hypothetical protein
SMb20949 exoV Putative pyruvyltransferase protein
SMb20954 exoH succinyltransferase protein
SMb20960 exoN UDPglucose pyrophosphorylase protein
SMc00062 Hypothetical protein
SMc00611 lpiA Transmembrane protein
SMc01505 Hypothetical protein
SMc01580 Hypothetical transmembrane protein
SMc01765 Hypothetical transmembrane protein
SMc01855 Hypothetical transmembrane protein
SMc01904 clpX Probable ATP-dependent CLP Protease ATP-binding subunit protein
SMc02052 Conserved Hypothetical protein
SMc02156 Conserved Hypothetical protein
SMc02365 degP1 Protease precursor protein
SMc02366 Putative transcription regulator protein
SMc02560 chvI transcriptionAL regulatorY protein
SMc02655 Hypothetical transmembrane protein
SMc04128 Putative heavy metal transporting ATPase protein
SMc04350 Putative multidrug efflux system transmembrane protein

CLUSTER H

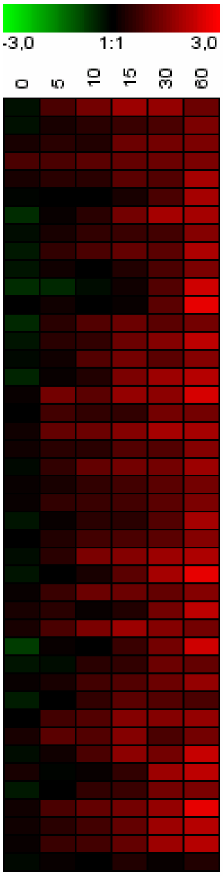

SMa0045	Cah Probable carbonic anhydrase
SMa0172	Conserved Hypothetical protein
SMa1078	Conserved Hypothetical protein
SMa1151	Conserved Hypothetical protein
SMb20359	Hypothetical protein
SMb20486	Putative sugar ABC transporter permease protein
SMb20611	dctA C4-dicarboxylate transport protein
SMb20944	exoQ Putative polysaccharide polymerase, similar to Wzy protein
SMb20946	exoY galactosyltransferase protein
SMb20955	exoK endo-beta-1,3-1,4-glycanase protein
SMb21440	Hypothetical protein
SMb21491	Hypothetical exported protein
SMb21516	Conserved Hypothetical protein
SMb21690	exoW glucosyltransferase protein
SMc00045	cycF Putative Cytochrome C signal peptide protein
SMc00063	Hypothetical transmembrane protein
SMc00103	dhe Putative Alpha-halocarboxylic acid dehalogenase protein
SMc00186	Putative ABC transporter ATP-binding transmembrane protein
SMc00346	Hypothetical transmembrane protein
SMc00610	Conserved Hypothetical protein
SMc01225	Putative transcription regulator protein
SMc01341	Hypothetical/unknown protein
SMc01428	cspA2 Probable cold shock transcription regulator protein
SMc01556	Conserved Hypothetical protein
SMc01764	ate Putative arginyl-tRNA protein transferase
SMc01769	Hypothetical protein
SMc01774	Hypothetical transmembrane protein
SMc01905	lon Probable ATP-dependent Protease LA protein
SMc02078	exoR Exopolysaccharide biosynthesis regulatorY protein
SMc02187	Putative Integrase DNA protein
SMc02278	Hypothetical unknown transmembrane protein
SMc02382	Conserved Hypothetical protein
SMc02491	Hypothetical protein
SMc03780	Hypothetical protein
SMc03783	ctpA Putative Carboxy-terminal processing protease precursor signal peptide protein
SMc03900	ndvA Beta-1-->2Glucan export ATP-binding protein
SMc03999	Hypothetical protein
SMc04167	Putative HIistidine-rich transporter transmembrane protein
SMc04246	Hypothetical transmembrane signal peptide protein
SMc04267	lpsS LPS sulfotransferase
SMc04459	ftsH Probable metalloprotease transmembrane protein
SMc04461	TolB protein precursor
SMc04865	Hypothetical protein

CLUSTER I

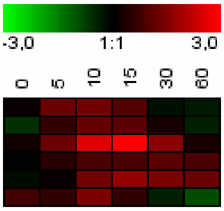

SMa1220	FixN1 Heme b / copper cytochrome c oxidase subunit
SMa1243	Azu1 pseudoazurin (blue copper protein)
SMc00641	serA Putative D-3-phosphoglycerate dehydrogenase protein
SMc01226	Putative transcription regulator protein
SMc02202	Hypothetical protein
SMc03290	Hypothetical protein

CLUSTER J

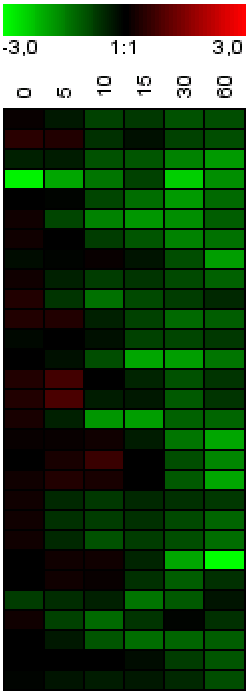

SMa0392	ABC transporter, periplasmic solute-binding protein
SMb20325	ThuE probable trehalosemaltose-binding protein
SMb20497	lyx Putative L-xylulose kinase protein
SMb20988	Conserved Hypothetical protein
SMb21176	phoD phosphate uptake ABC transporter periplasmic solute-binding protein precursor
SMb21177	phoC phosphate uptake ABC transporter ATP-binding protein
SMc00094	betB Betaine aldehyde dehydrogenase BADH oxireductese NAD protein
SMc00159	Hypothetical signal peptide protein
SMc00638	Putative heat resistant agglutinin 1 protein
SMc02145	Hypothetical signal peptide protein
SMc02146	Putative Phosphate-binding periplasmic protein
SMc02396	Probable Outer membrane protein
SMc02400	Probable Outer membrane protein
SMc02479	mdh Probable Malate dehydrogenase protein
SMc02480	sucC Probable Succinyl-coA synthetase beta chain protein
SMc02582	Conserved Hypothetical protein
SMc03027	flgB flagellar basal-body rod protein
SMc03029	fliE flagellar hook-basal body complex protein
SMc03030	flgG flagellar basal-body rod protein
SMc03037	flaA flagellin A protein
SMc03038	flaB flagellin B protein
SMc03039	flaD Probable flagellin D protein
SMc03049	flgL Putative flagellar hook-associated protein
SMc03050	flaF Putative flagellin synthesis regulator protein
SMc03138	Putative Sugar kinase protein
SMc03806	glnK Probable Nitrogen regulatorY protein PII 2
SMc04009	Conserved Hypothetical protein
SMc04059	Hypothetical protein
SMc04111	cpaC1 Putative pilus assembly transmembrane protein

CLUSTER K

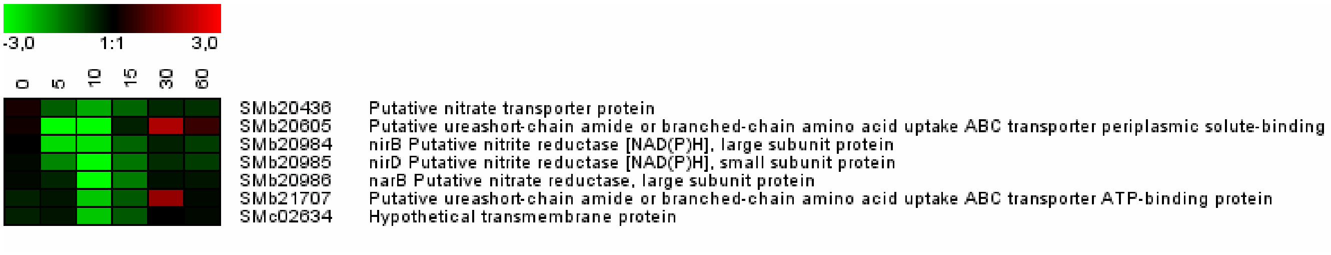
-3,0
1:1
3,0
0
5
10
15
30
60
SMb20436 Putative nitrate transporter protein
SMb20605 Putative ureashort-chain amide or branched-chain amino acid uptake ABC transporter periplasmic solute-binding
SMb20984 nirB Putative nitrite reductase [NAD(P)H], large subunit protein
SMb20985 nirD Putative nitrite reductase [NAD(P)H], small subunit protein
SMb20986 narB Putative nitrate reductase, large subunit protein
SMb21707 Putative ureashort-chain amide or branched-chain amino acid uptake ABC transporter ATP-binding protein
SMc02634 Hypothetical transmembrane protein

CLUSTER L

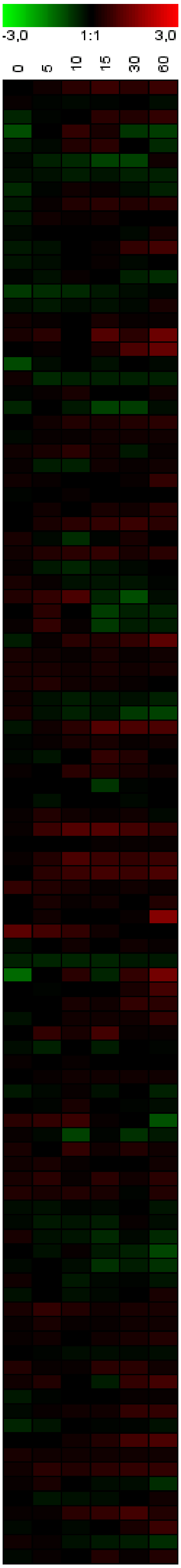

SMa0473	Conserved Hypothetical protein
SMa0994	Hypothetical protein
SMa1079	TspO Tryptophan rich sensory protein homologue
SMa1082	Hypothetical protein
SMa1182	NosZ N2O reductase
SMa1750	Hypothetical protein
SMa1898	Hypothetical protein
SMa1961	Putative Polyhydrixyalkanoate depolymerase
SMb20696	Hypothetical protein
SMb20707	cyaG2 Putative adenylate cyclase protein
SMb20777	TRm19 Putative transposase of insertion sequence ISRm19 protein
SMb20956	exoL Putative glucosyltransferase protein
SMb21236	Putative ATPGTP-binding protein
SMb21259	Hypothetical exported protein precursor
SMb21295	Putative small heat shock protein, hsp20 family
SMb21566	groEL5 Putative heat shock protein groEL
SMc00043	sodB Superoxide dismutase Fe protein
SMc00070	Conserved Hypothetical signal peptide protein
SMc00115	Conserved Hypothetical protein
SMc00276	Conserved Hypothetical protein
SMc00283	Putative transcription regulator protein
SMc00301	sufA Putative FeS assembly scaffold
SMc00302	Conserved Hypothetical protein
SMc00329	irr Putative Iron response regulator protein
SMc00341	Hypothetical transmembrane protein
SMc00565	rplI Probable 50S ribosomal protein L9
SMc00591	Hypothetical/unknown signal peptide protein
SMc00810	hypothetical protein
SMc00827	Putative transport transmembrane protein
SMc00897	pmbA Hypothetical PMBA protein
SMc00914	Putative oxidoreductase protein
SMc00949	Conserved Hypothetical protein
SMc01107	Conserved Hypothetical protein
SMc01142	grpE Probable heat shock protein
SMc01224	trxB Probable Thioredoxin reductase protein
SMc01309	rplC Probable 50S ribosomal protein L3
SMc01311	tufA Probable elongation factor TU protein
SMc01326	tufB Probable elongation factor TU protein
SMc01365	rnr Putative Exoribonuclease II protein
SMc01440	hflC Putative hydrolase serine protease transmembrane protein
SMc01441	hflK Putative membrane bound Protease protein
SMc01518	Conserved Hypothetical protein
SMc01578	aatA Aspartate aminotransferase A (transaminase) protein
SMc01719	mcpT chemoreceptor transmembrane protein
SMc01788	Hypothetical protein
SMc01813	Conserved Hypothetical protein
SMc01815	Putative oxidoreductase Iron-sulfur protein
SMc01832	ureE Putative Urease accessory protein
SMc01848	Conserved Hypothetical protein
SMc01860	ftsI Probable Penicillin-binding transmembrane protein
SMc01947	Conserved Hypothetical transmembrane protein
SMc02075	Conserved Hypothetical protein
SMc02106	Conserved Hypothetical protein
SMc02109	clpA Probable ATP-dependent CLP Protease ATP-binding subunit protein
SMc02110	ATP-dependent Clp protease adapter protein clpS1
SMc02139	Hypothetical protein
SMc02151	Hypothetical virulence associated protein homologue
SMc02275	pncA Probable Pyrazinamidase/nicotinamidase protein
SMc02284	Hypothetical signal peptide protein
SMc02390	gst7 Putative Glutathione S-transferase protein
SMc02392	Hypothetical protein
SMc02403	Putative Murein transglycosylase protein
SMc02433	clpB Probable ATP-dependent Protease (heat shock protein)
SMc02435	hemK1 Putative Methyltransferase protein
SMc02443	grxC Probable Glutaredoxin 3 protein
SMc02518	Putative ATP-binding ABC transporter protein
SMc02575	hslV Probable heat shock protein
SMc02576	Hypothetical Acetyltransferase protein
SMc02720	clpP2 CLP Protease Proteolytic subunit protein
SMc02728	fhs Probable Formate-tetrahydrofolate ligase protein
SMc02735	Hypothetical protein
SMc02755	ahcY Probable Adenosylhomocysteinase protein
SMc02820	cpaF1 Putative pilus assembly protein
SMc02885	msrA1 Probable peptide Methionine sulfoxide reductase protein
SMc02898	kdsB Probable 3-deoxy-manno-octulosonate cytidylyltransferase (CMP-KDO synthetase) protein
SMc02978	Conserved Hypothetical protein
SMc02980	Hypothetical protein
SMc03000	Putative permease ABC transporter protein
SMc03016	Putative transcription regulator protein
SMc03046	Putative transcription regulator protein
SMc03047	flgE flagellar hook protein
SMc03064	aglA Probable alpha-glucosidase protein
SMc03090	cheW3 Putative chemotaxis protein
SMc03105	dxr Probable 1-deoxy-D-xylulose 5-phosphate reductoisomerase protein
SMc03111	pmi Mannose-6-phosphate isomerase protein
SMc03151	Conserved Hypothetical protein
SMc03152	Hypothetical transmembrane protein
SMc03205	purU1 Putative Formyltetrahydrofolate deformylase protein
SMc03245	Putative Amidase protein
SMc03773	Conserved Hypothetical protein
SMc03784	Hypothetical transmembrane protein
SMc03802	Conserved Hypothetical protein
SMc03819	Conserved Hypothetical protein
SMc03836	tesA Putative Acyl-coA Thioesterase I protein
SMc03838	Hypothetical transmembrane protein
SMc03893	Putative Amino-acid transport system permease ABC transporter protein
SMc04026	gltD Probable glutamate synthase small chain protein
SMc04040	ibpA Heat shock protein
SMc04091	htpX Putative Protease transmembrane protein
SMc04213	dgkA Diacylglycerol kinase protein
SMc04346	ilvC ketol-acid reductoisomerase protein
SMc04865	Hypothetical protein
