# Supplementary material for: The role of sigma factor RpoH1 in the pH stress response of Sinorhizobium meliloti
Source: BMC Microbiol. 2010 Oct 18;10:265. doi: 10.1186/1471-2180-10-265 (PMC2976971; doi:10.1186/1471-2180-10-265)
Supplement: Additional file 7 — Quantitative RT-PCR. qRT-PCR was performed for validation of the microarray expression data. The six genes used in the experiment were smb20611, smc01505, grpE, lpiA, exoY and mcpT. Differences in gene expression were determined by comparing the crossing points of samples measured in three replicates. Comparison of expression data was always performed between samples transferred to medium at pH 5.75 and control samples transferred to control medium at pH 7.0, 10 or 60 minutes after pH shift. In the group of genes analyzed, RpoH1-dependent, RpoH1-independent and complex regulation could be observed, in accordance to the microarray expression data. Section A includes the results obtained by qRT-PCR. The M-values of the microarray were included in section B to facilitate the comparison. [file 1471-2180-10-265-S7.PDF]

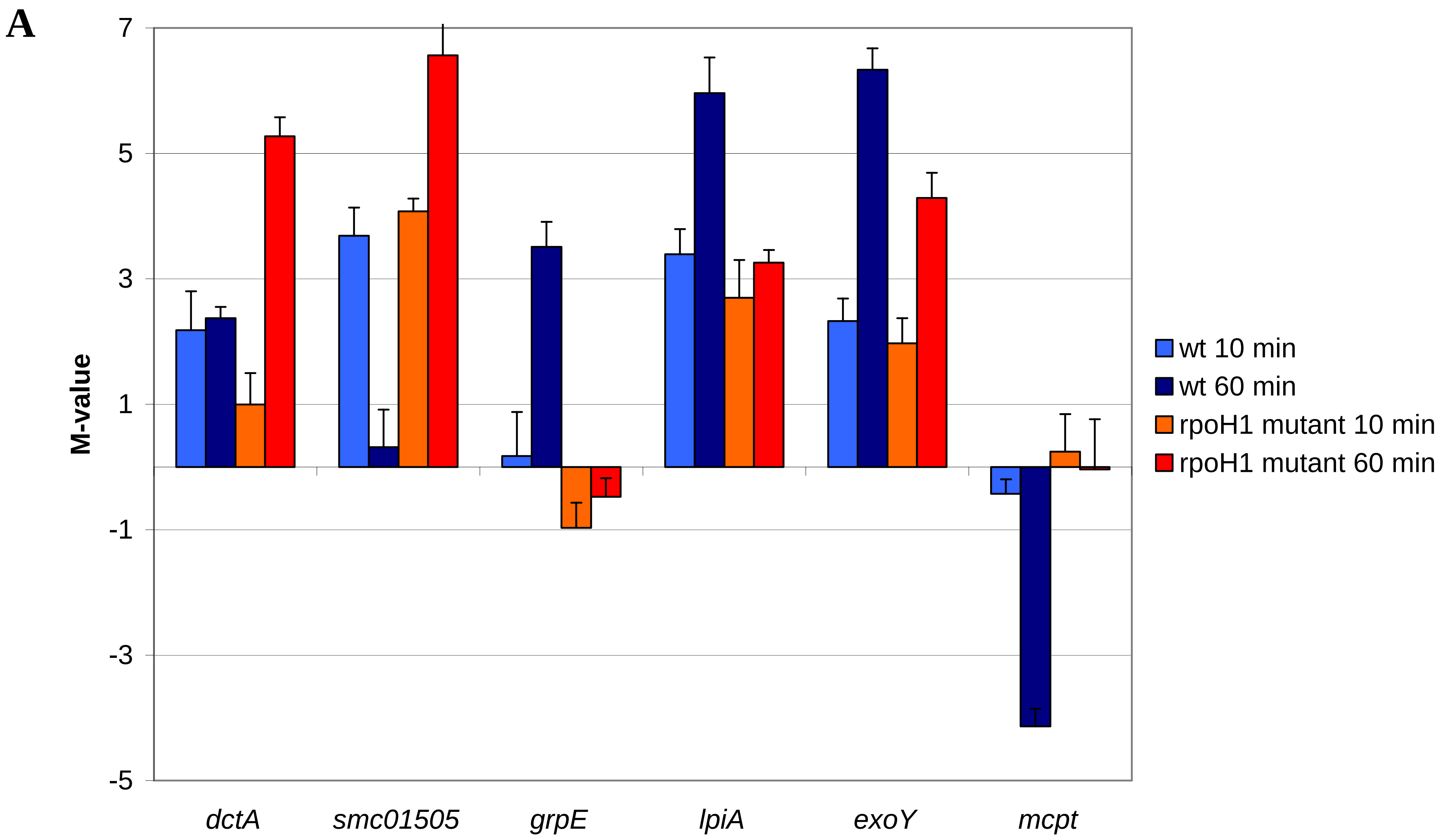

Additional file 7. Validation of microarray data by quantitative RT-PCR. (A) M-values obtained by qRT-PCR for the six selected genes: *dctA*, *smc01505*, *grpE*, *lpiA, exoY* and *mcpT*. Comparison of expression data was performed between samples transferred to medium at pH 5.75 and control samples transferred to medium at pH 7, at time points 10 or 60 minutes after pH shift. Error bars indicate standard deviation calculated from three independent cultures.

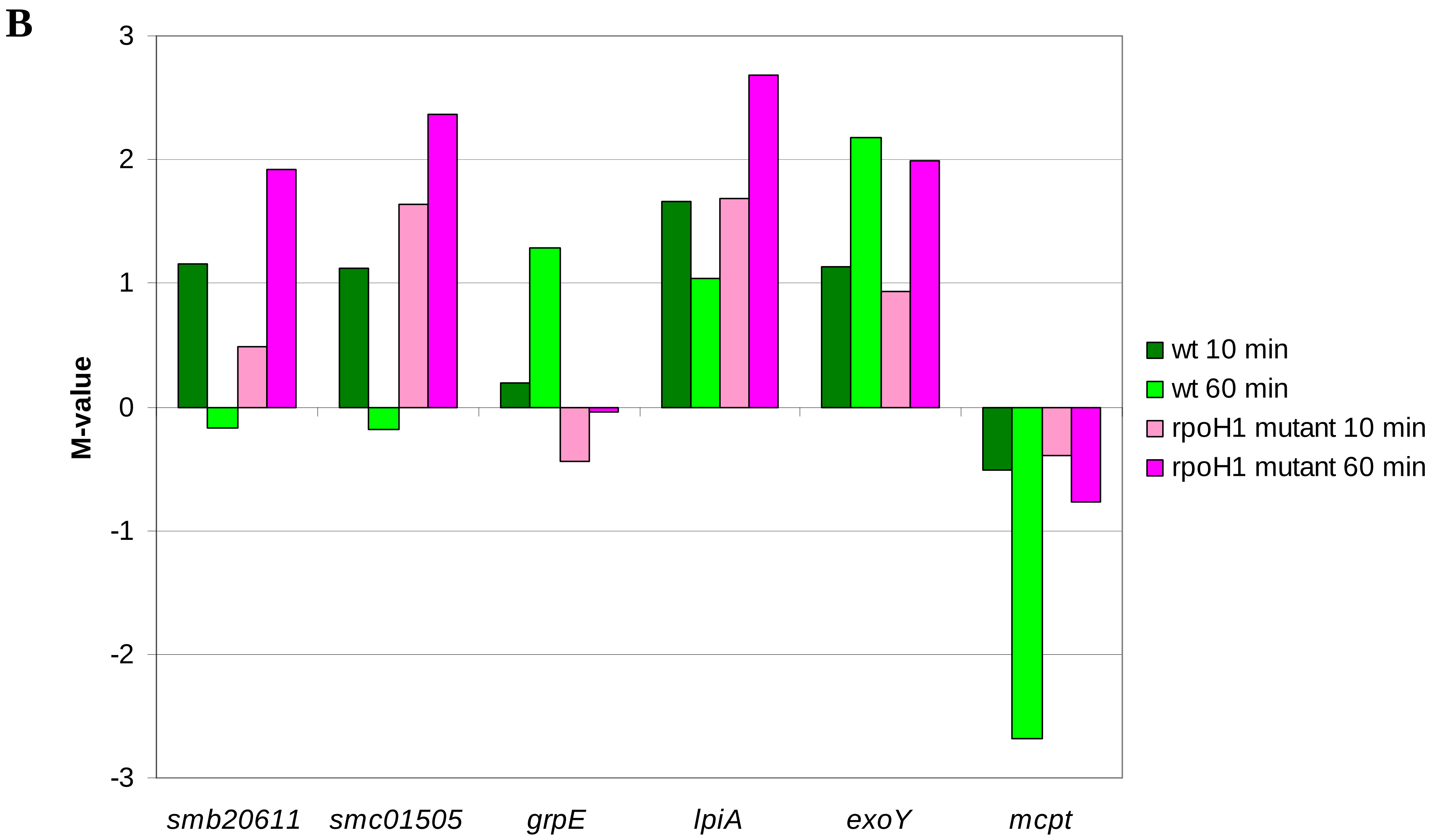

Additional file 7. Validation of microarray data by quantitative RT-PCR. (B) M-values obtained in the microarray analyses of the six selected genes: *dctA*, *smc01505*, *grpE*, *lpiA, exoY* and *mcpT*. Comparison of expression data was performed between samples transferred to medium at pH 5.75 and control samples transferred to medium at pH 7, at time points 10 or 60 minutes after pH shift.
